# Supplementary figures and images for: Auto-inhibition of PRC2 by the broadly expressed long isoform of AEBP2
Source: EMBO J. 2025 Oct 30;44(23):6979–7020. doi: 10.1038/s44318-025-00616-9 (PMC12669776; doi:10.1038/s44318-025-00616-9)

Mw kDa

Mw PRC2 PRC2-AEBP2<sup>L(iso2)</sup>  
PRC2-AEBP2<sup>S</sup>

148—  
98—  
64—  
50—  
36—

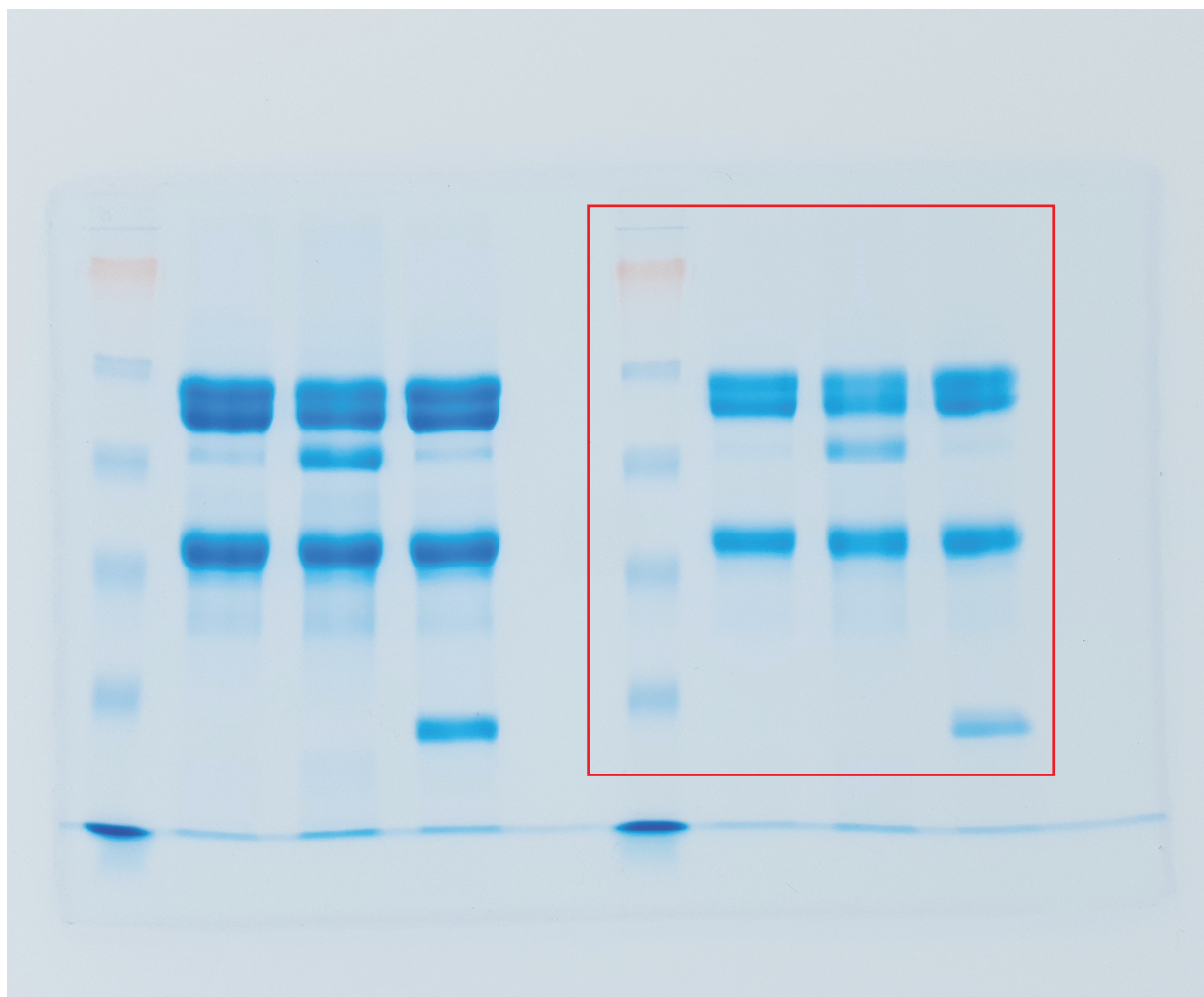

Supplement: Supplementary file 7 — Source data Fig. 1 [file 44318_2025_616_MOESM7_ESM.zip › Figure 1/1b/1b.pdf]

PRC2

PRC2-AEBP2<sup>L(iso1)</sup>

2x

2x

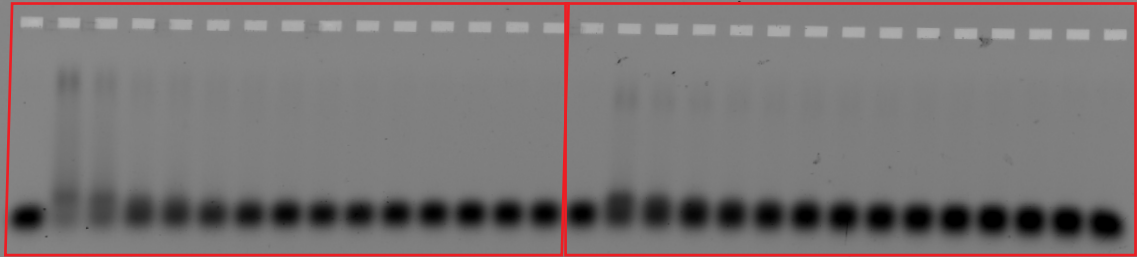

PRC2-AEBP2<sup>L(iso2)</sup>

PRC2-AEBP2<sup>S</sup>

2x

2x

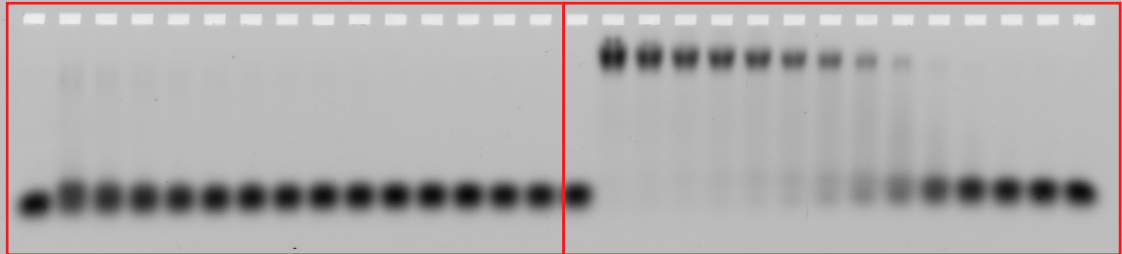

Supplement: Supplementary file 7 — Source data Fig. 1 [file 44318_2025_616_MOESM7_ESM.zip › Figure 1/1c/1c.pdf]

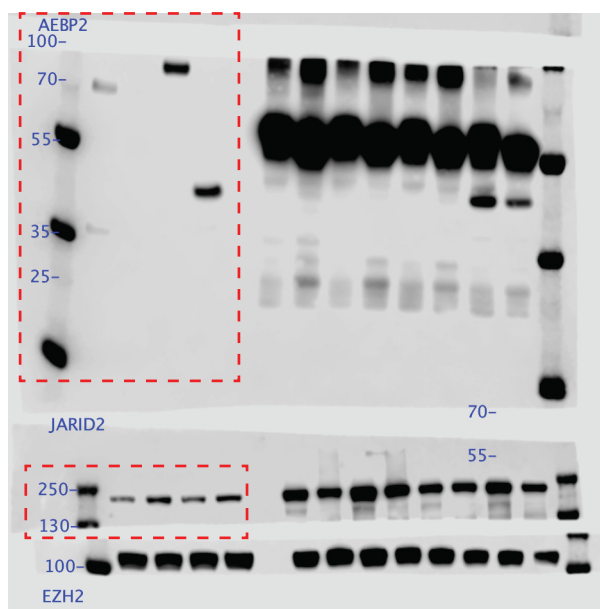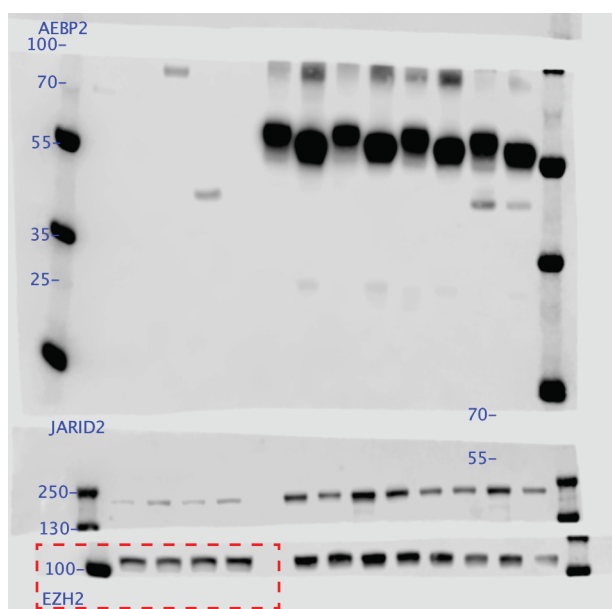

Supplement: Supplementary file 8 — Source data Fig. 2 [file 44318_2025_616_MOESM8_ESM.zip › Figure 2/2B/Source Data Fig 2B.pdf]

Uncropped gels for Fig. 3

AEBP2

kDa

100  
70  
55  
35

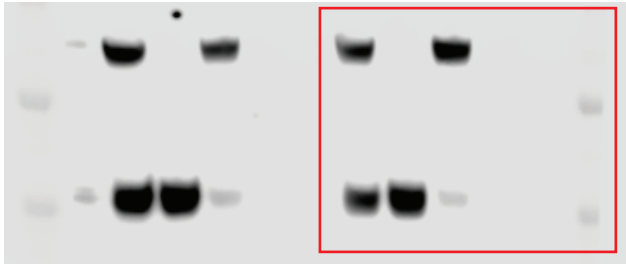

SUZ12

kDa

250  
130  
100

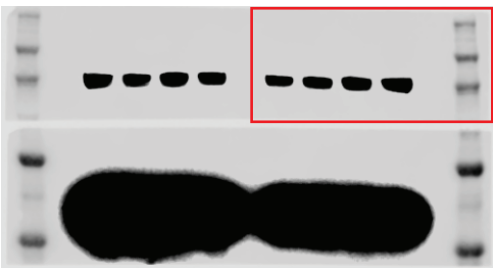

JARID2

kDa

250  
130  
100

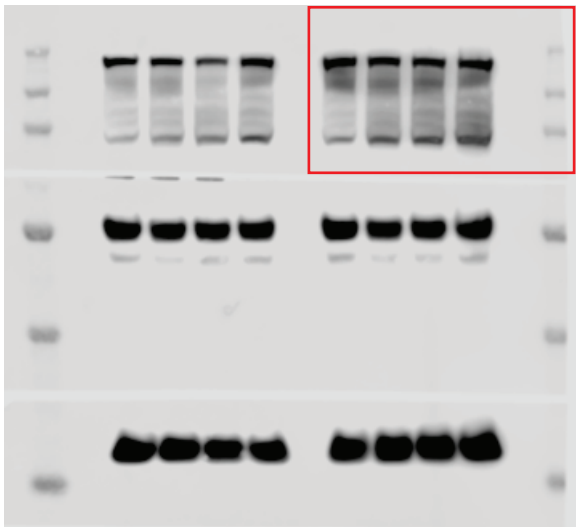

GAPDH

kDa

55 35

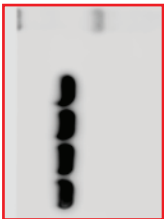

MTF2

kDa

100  
70  
55

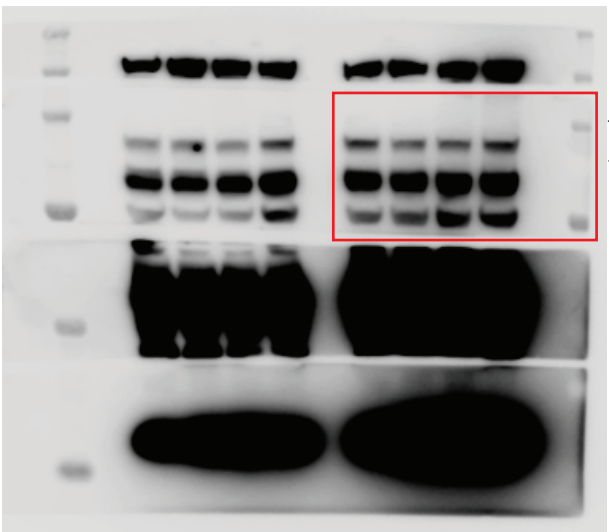

EPOP

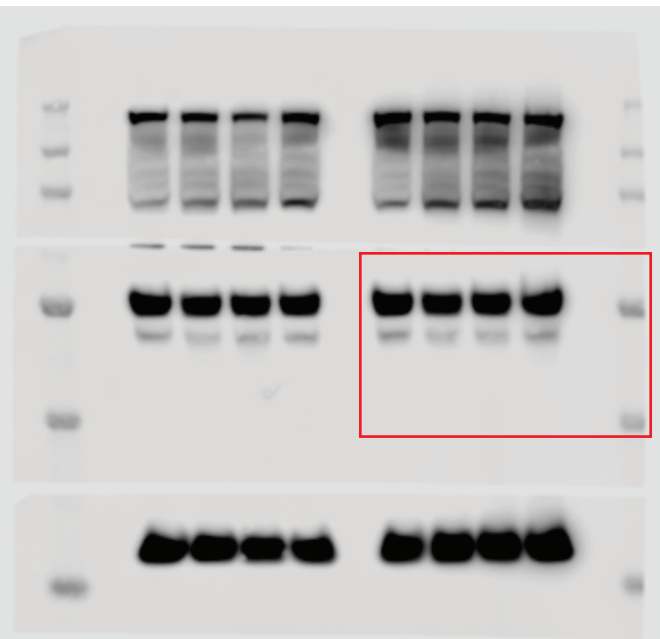

Supplement: Supplementary file 9 — Source data Fig. 3 [file 44318_2025_616_MOESM9_ESM.zip › Figure 3/3G/3G.pdf]

# PRC2-AEBP2<sup>L(TEV)</sup>-JARID2

TEV: + -

Mw (kDa)  
198—  
98—  
62—  
49—  
38—  
28—  
17—  
14—

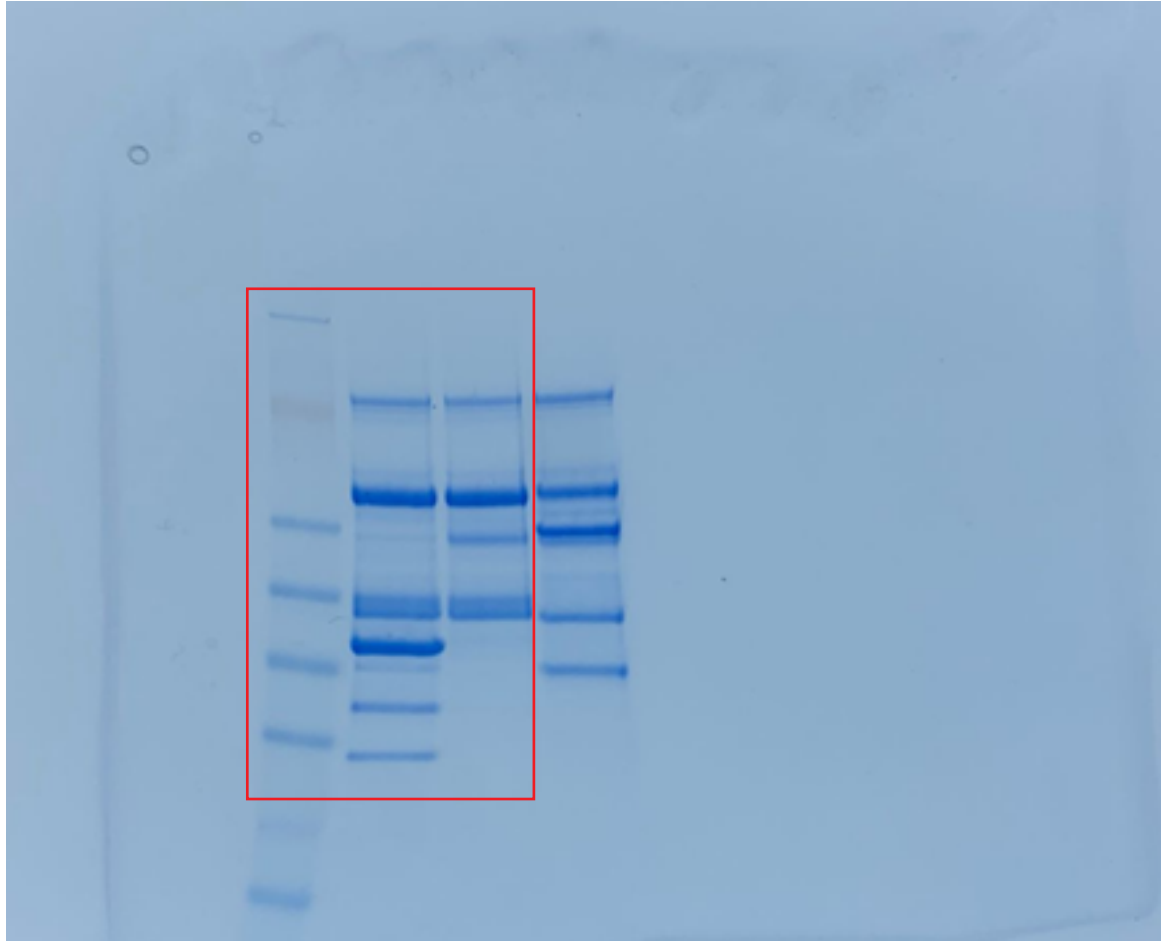

Supplement: Supplementary file 11 — Source data Fig. 6 [file 44318_2025_616_MOESM11_ESM.zip › Figure 6/6b/6b.pdf]

# PRC2-AEBP2-JARID2

L

S

Mw (kDa)

mt1K2K  
mt1A2A  
mt1Δ2Δ

Mw (kDa)

198—

98—

62—

49—

38—

28—

17—

14—

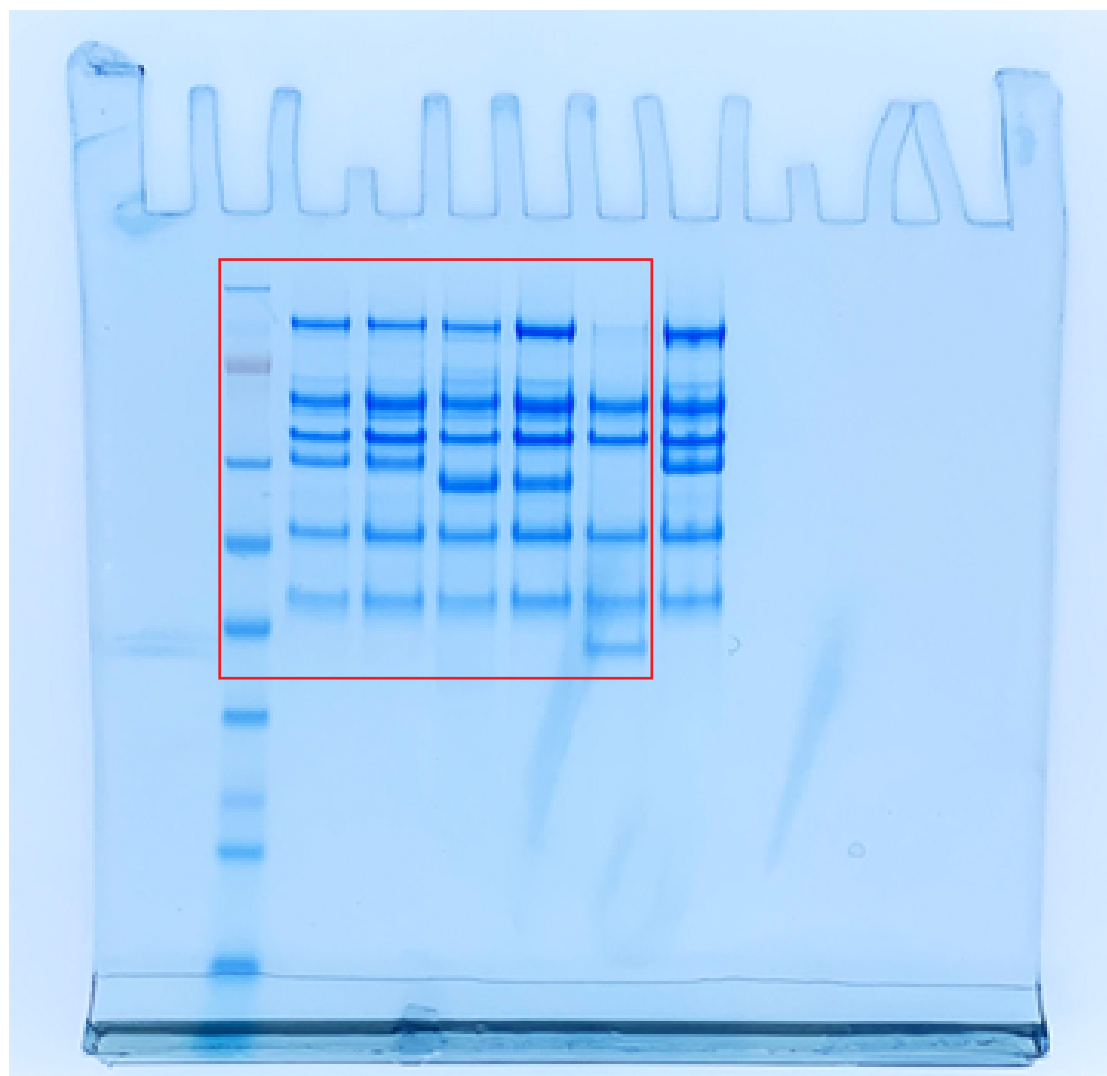

Supplement: Supplementary file 11 — Source data Fig. 6 [file 44318_2025_616_MOESM11_ESM.zip › Figure 6/6e/6e.pdf]

PRC2-AEBP2<sup>L</sup>-JARID2      WT      mt1A2A      mt1Δ2Δ  
[nM]:   -   180   60   20   180   60   20   180   60   20

H3K27me3

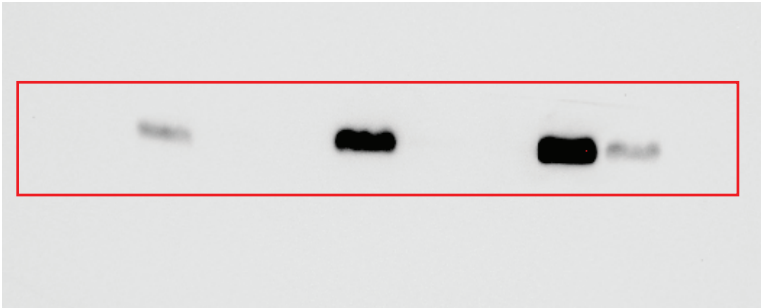

H3K27me2

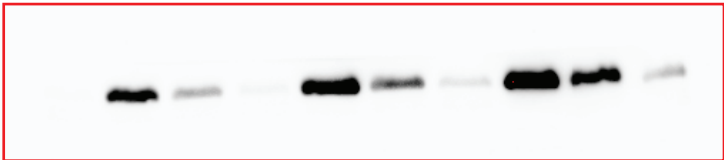

H3K27me1

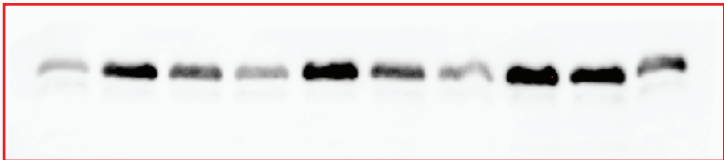

H3

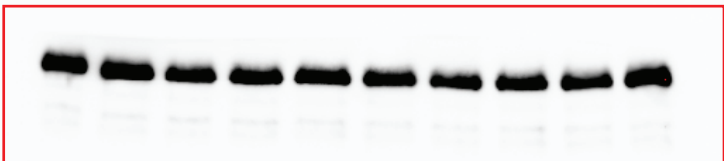

SUZ12

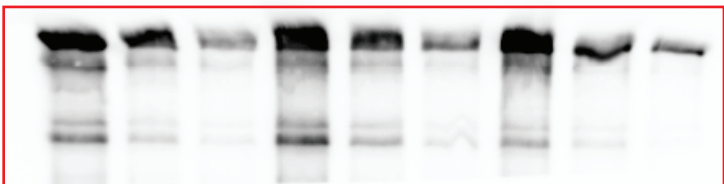

Supplement: Supplementary file 11 — Source data Fig. 6 [file 44318_2025_616_MOESM11_ESM.zip › Figure 6/6g/6g.pdf]

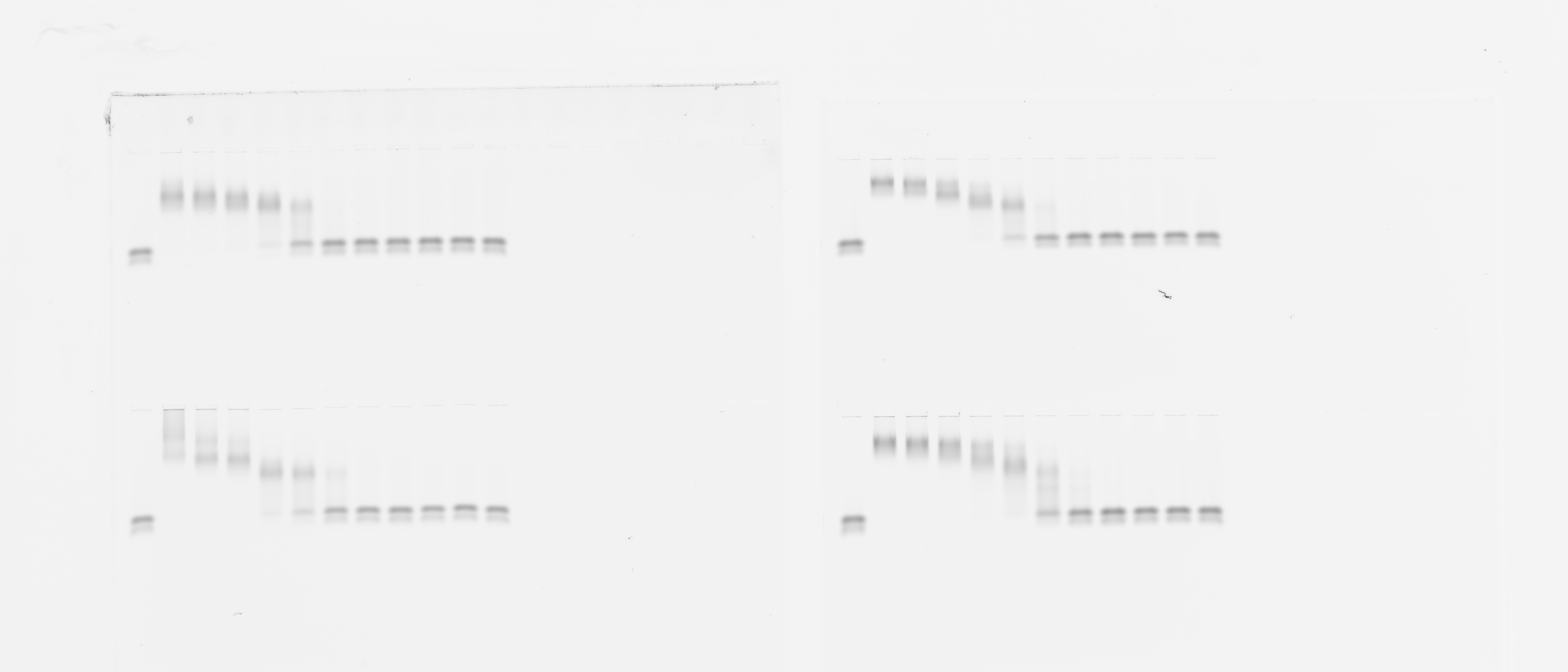

Supplement: Supplementary file 11 — Source data Fig. 6 [file 44318_2025_616_MOESM11_ESM.zip › Figure 6/6h/6h.gel]
