# Supplementary material for: Auto-inhibition of PRC2 by the broadly expressed long isoform of AEBP2
Source: EMBO J. 2025 Oct 30;44(23):6979–7020. doi: 10.1038/s44318-025-00616-9 (PMC12669776; doi:10.1038/s44318-025-00616-9)
Supplement: Supplementary file 10 — Source data Fig. 5 [file 44318_2025_616_MOESM10_ESM.zip › Figure 5/5g/5g.pdf]

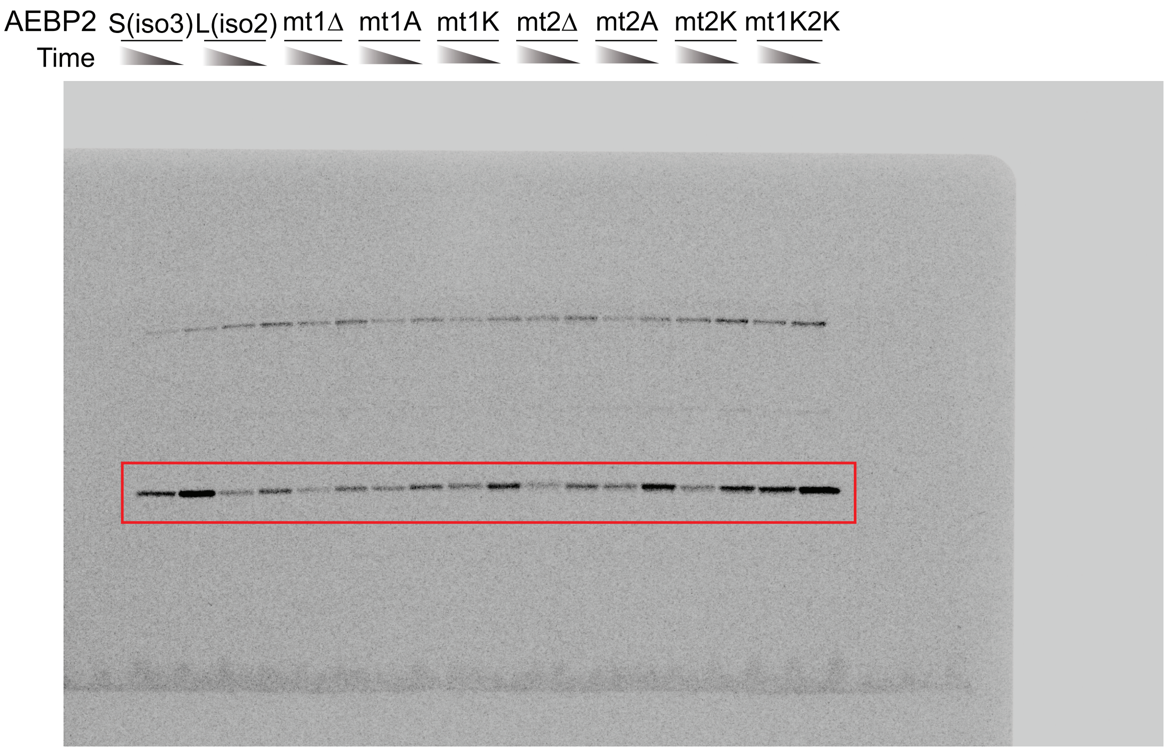
AEBP2
Time
S(iso3)
L(iso2)
mt1Δ
mt1A
mt1K
mt2Δ
mt2A
mt2K
mt1K2K

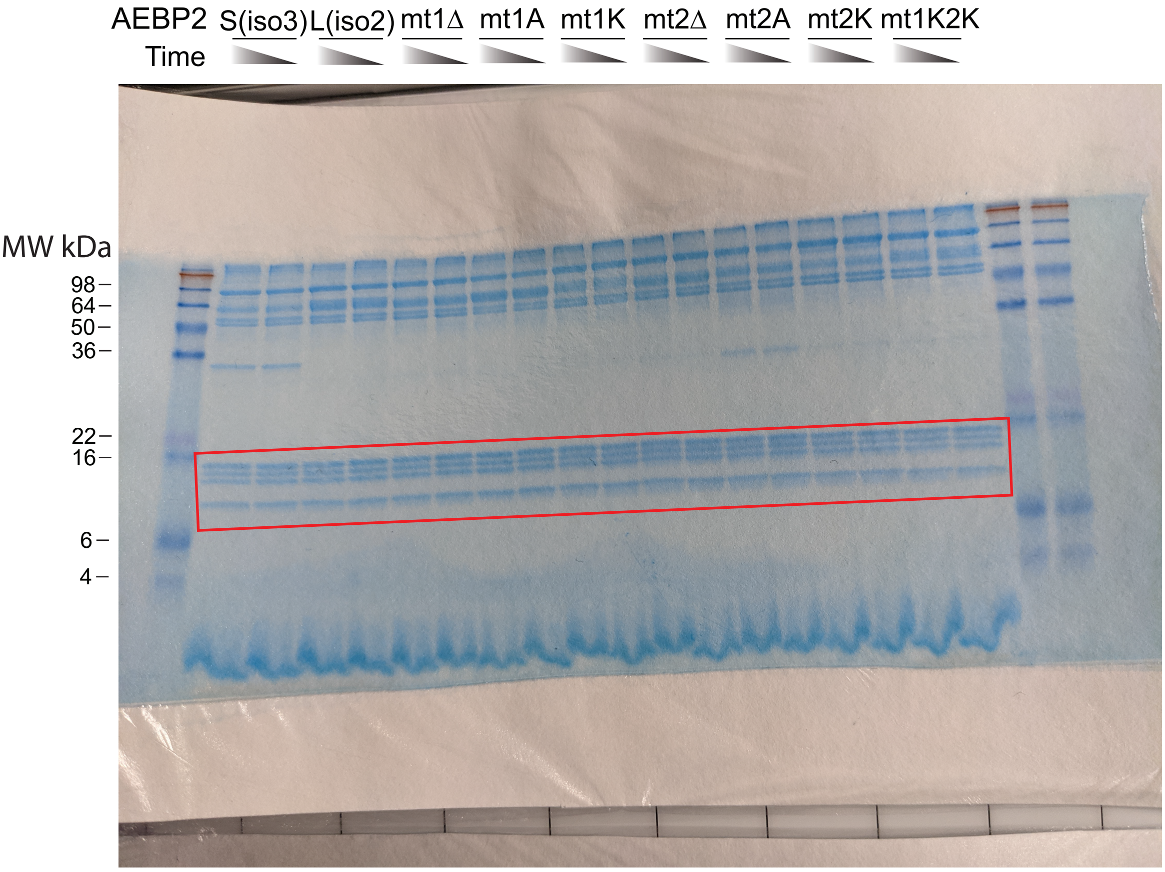
AEBP2
Time
S(iso3)
L(iso2)
mt1Δ
mt1A
mt1K
mt2Δ
mt2A
mt2K
mt1K2K
MW kDa
98
64
50
36
22
16
6
4
