# Supplementary material for: Auto-inhibition of PRC2 by the broadly expressed long isoform of AEBP2
Source: EMBO J. 2025 Oct 30;44(23):6979–7020. doi: 10.1038/s44318-025-00616-9 (PMC12669776; doi:10.1038/s44318-025-00616-9)
Supplement: Supplementary file 11 — Source data Fig. 6 [file 44318_2025_616_MOESM11_ESM.zip › Figure 6/6h/6h.pdf]

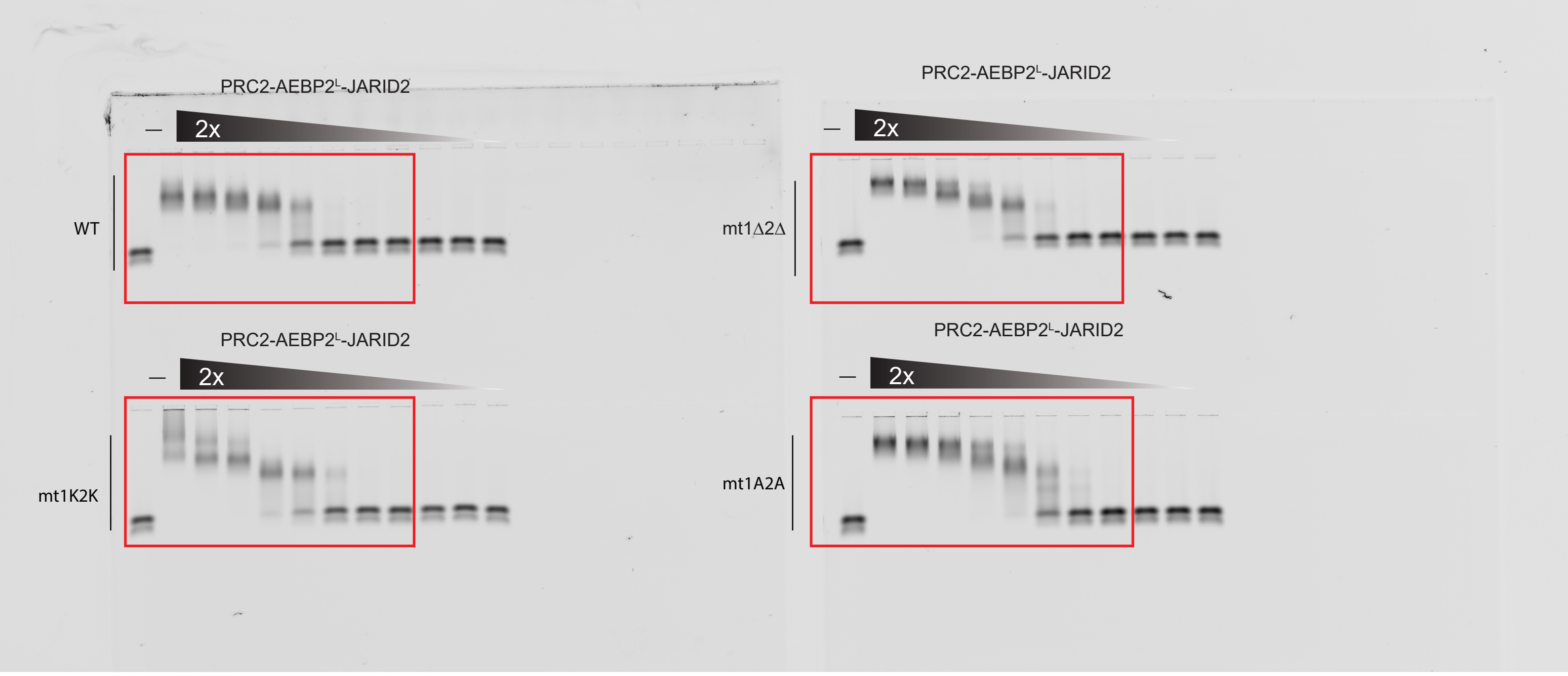

PRC2-AEBP2L-JARID2
2x
WT
mt1Δ2Δ
mt1K2K
mt1A2A
