# Supplementary material for: Auto-inhibition of PRC2 by the broadly expressed long isoform of AEBP2
Source: EMBO J. 2025 Oct 30;44(23):6979–7020. doi: 10.1038/s44318-025-00616-9 (PMC12669776; doi:10.1038/s44318-025-00616-9)
Supplement: Supplementary file 12 — Expanded View Figures [file 44318_2025_616_MOESM12_ESM.pdf]

# Expanded View Figures

**Figure EV1. AEBP2$^{L}$ inhibits the DNA-binding activity of PRC2 in vitro.**

(**A**) Multiple sequence alignment of human AEBP2 isoforms AEBP2$^{L(Iso1)}$ (Uniprot: Q6ZN18-1), AEBP2$^{L(Iso2)}$ (Uniprot: Q6ZN18-2), and AEBP2$^{S}$ (Uniprot: Q6ZN18-3), performed using Clustal O (1.2.4). (**B**) EMSA performed with 0.7% agarose and used to quantify the affinity of the indicated PRC2 complexes for fluorescein-labelled DNA in Fig. 1C. (**C**) Fluorescence anisotropy assays used to quantify the affinity of the indicated PRC2 complexes for the same fluorescein-labelled DNA probe used for EMSA in Fig. 1C. Three sets of assays were performed using the same binding buffer but with differing levels of KCl in the binding buffer as indicated above each graph (25/50/100 mM KCl). Data represent the mean of three independent experiments that were carried out on different days and error bars represent standard deviation.

© The Author(s) 

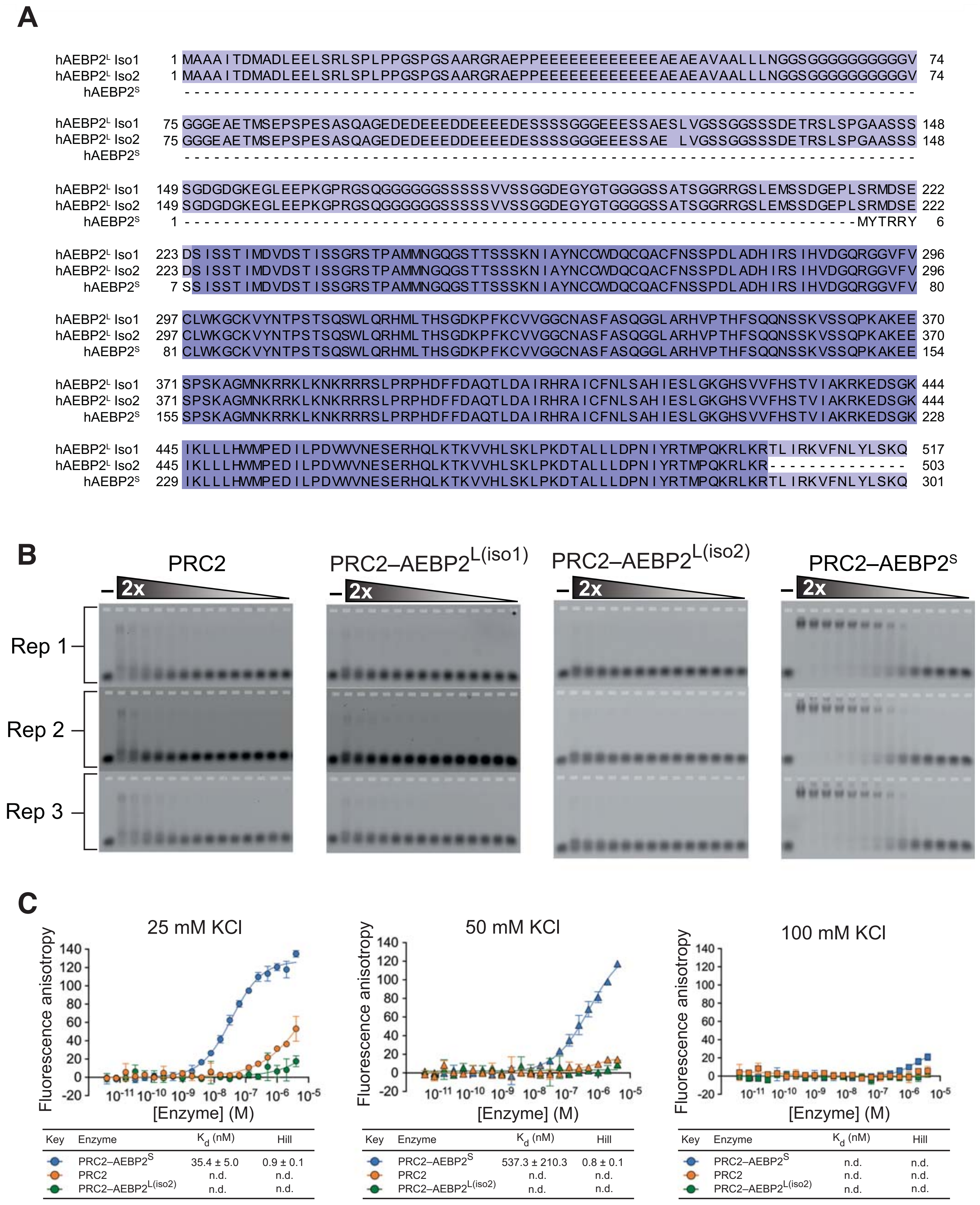
A
hAEBP2$^{L}$ Iso1 1 MAAAITDMADLEELSRLSPLPPGSPGSAARGRAEPPEEEEEEEEEEEEAEAEAVAALLLNGGSGGGGGGGGGGV 74
hAEBP2$^{L}$ Iso2 1 MAAAITDMADLEELSRLSPLPPGSPGSAARGRAEPPEEEEEEEEEEEEAEAEAVAALLLNGGSGGGGGGGGGGV 74
hAEBP2$^{S}$ ---------------------------------------------------------------------------
hAEBP2$^{L}$ Iso1 75 GGGEAETMSEPSPESASQAGEDEDEEEDDEEEEDESSSSGGGEEESSAESLVGSSGGSSSDETRSLSPGAASSS 148
hAEBP2$^{L}$ Iso2 75 GGGEAETMSEPSPESASQAGEDEDEEEDDEEEEDESSSSGGGEEESSAE LVGSSGGSSSDETRSLSPGAASSS 148
hAEBP2$^{S}$ ---------------------------------------------------------------------------
hAEBP2$^{L}$ Iso1 149 SGDGDGKEGLEEPKGPRGSQGGGGGGGSSSSSVVSSGGDEGYGTGGGGSSATSGGRRGSLEMSSDGEPLSRMDSE 222
hAEBP2$^{L}$ Iso2 149 SGDGDGKEGLEEPKGPRGSQGGGGGGGSSSSSVVSSGGDEGYGTGGGGSSATSGGRRGSLEMSSDGEPLSRMDSE 222
hAEBP2$^{S}$ 1 --------------------------------------------------------------------MYTRRY 6
hAEBP2$^{L}$ Iso1 223 DSISSTIMDVDSTISSGRSTPAMMNGQGSTTSSSKNIAYNCCWDQCQACFNSSPDLADHIRSIHVDGQRGGVFV 296
hAEBP2$^{L}$ Iso2 223 DSISSTIMDVDSTISSGRSTPAMMNGQGSTTSSSKNIAYNCCWDQCQACFNSSPDLADHIRSIHVDGQRGGVFV 296
hAEBP2$^{S}$ 7 SSISSTIMDVDSTISSGRSTPAMMNGQGSTTSSSKNIAYNCCWDQCQACFNSSPDLADHIRSIHVDGQRGGVFV 80
hAEBP2$^{L}$ Iso1 297 CLWKGCKVYNTPSTSQSWLQRHMLTHSGDKPFKCVVGGCNASFASQGGLARHVPTHFSQQNSSKVSSQPKAKEE 370
hAEBP2$^{L}$ Iso2 297 CLWKGCKVYNTPSTSQSWLQRHMLTHSGDKPFKCVVGGCNASFASQGGLARHVPTHFSQQNSSKVSSQPKAKEE 370
hAEBP2$^{S}$ 81 CLWKGCKVYNTPSTSQSWLQRHMLTHSGDKPFKCVVGGCNASFASQGGLARHVPTHFSQQNSSKVSSQPKAKEE 154
hAEBP2$^{L}$ Iso1 371 SPSKAGMNKRRKLKNKRRRSLPRPHDFFDAQTLDAIRHRAICFNLSAHIESLGKGHSVVFHSTVIAKRKEDSGK 444
hAEBP2$^{L}$ Iso2 371 SPSKAGMNKRRKLKNKRRRSLPRPHDFFDAQTLDAIRHRAICFNLSAHIESLGKGHSVVFHSTVIAKRKEDSGK 444
hAEBP2$^{S}$ 155 SPSKAGMNKRRKLKNKRRRSLPRPHDFFDAQTLDAIRHRAICFNLSAHIESLGKGHSVVFHSTVIAKRKEDSGK 228
hAEBP2$^{L}$ Iso1 445 IKLLLHWMPEDILPDWVVNESERHQLKTKVVHLSKLPKDTALLLDPNIYRTMPQKRLKRTLIRKVFNLYLSKQ 517
hAEBP2$^{L}$ Iso2 445 IKLLLHWMPEDILPDWVVNESERHQLKTKVVHLSKLPKDTALLLDPNIYRTMPQKRLKR-------------- 503
hAEBP2$^{S}$ 229 IKLLLHWMPEDILPDWVVNESERHQLKTKVVHLSKLPKDTALLLDPNIYRTMPQKRLKRTLIRKVFNLYLSKQ 301
B
PRC2
PRC2–AEBP2$^{L(iso1)}$
PRC2–AEBP2$^{L(iso2)}$
PRC2–AEBP2$^{S}$
– 2x
Rep 1
Rep 2
Rep 3
C
25 mM KCl
50 mM KCl
100 mM KCl
Fluorescence anisotropy
[Enzyme] (M)
Key Enzyme $K_d$ (nM) Hill
PRC2–AEBP2$^{S}$ 35.4 ± 5.0 0.9 ± 0.1
PRC2 n.d. n.d.
PRC2–AEBP2$^{L(iso2)}$ n.d. n.d.
PRC2–AEBP2$^{S}$ 537.3 ± 210.3 0.8 ± 0.1
PRC2 n.d. n.d.
PRC2–AEBP2$^{L(iso2)}$ n.d. n.d.
PRC2–AEBP2$^{S}$ n.d. n.d.
PRC2 n.d. n.d.
PRC2–AEBP2$^{L(iso2)}$ n.d. n.d.

 © The Author(s)

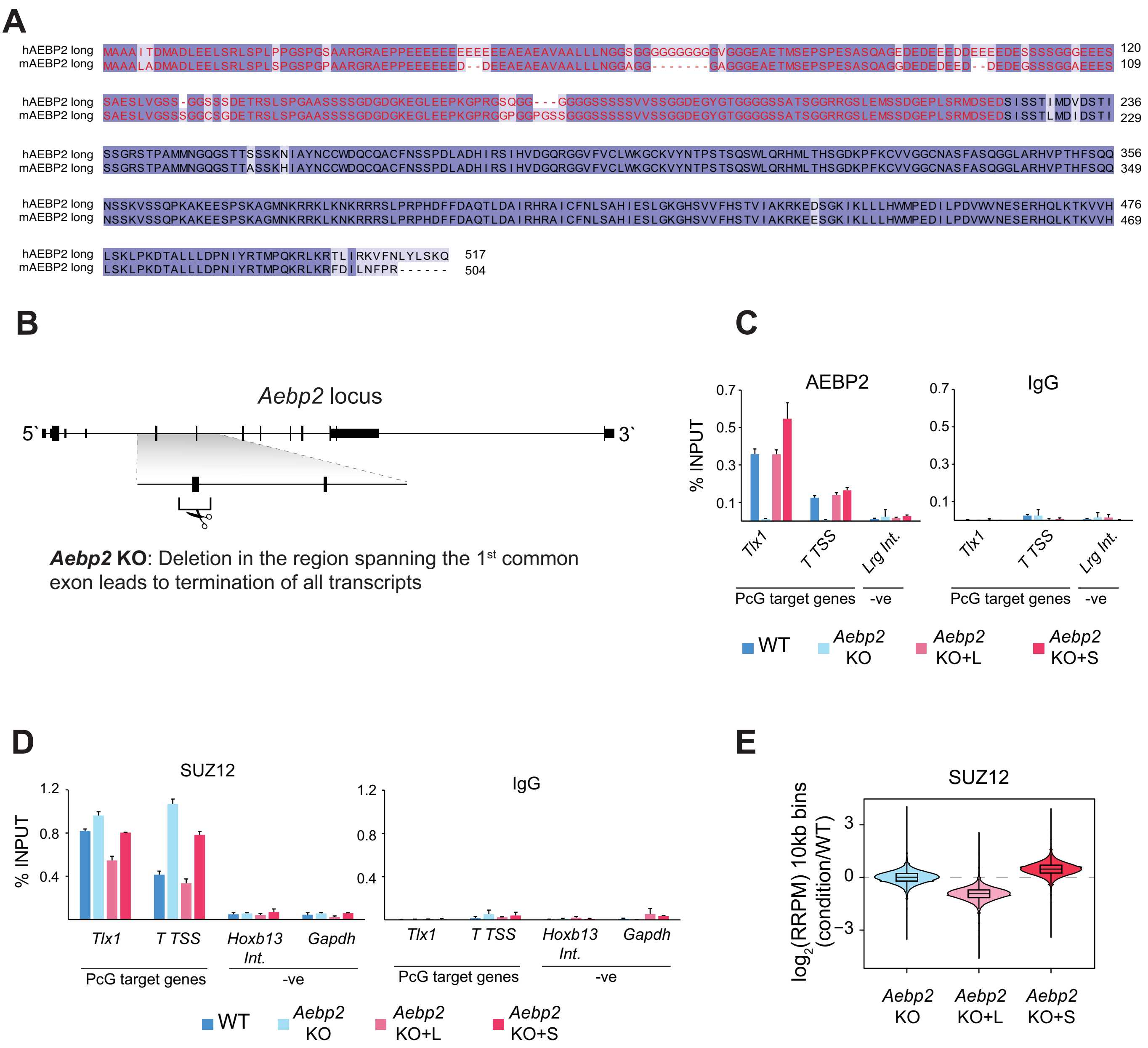


**Figure EV2. Ectopic expression of AEBP2[L] leads to reduced PRC2 binding to target genes in mouse embryonic stem cells.**

(**A**) Multiple sequence alignment of human AEBP2 long isoform (Uniprot: Q6ZN18-1) and mouse AEBP2 long isoform (Uniprot: Q9Z248-1), performed using Clustal O (1.2.4). (**B**) Schematic representation of the *Aebp2* gene locus depicting the positions of the sgRNAs used to generate the *Aebp2* KO cell line. See Dataset EV1 for sgRNA sequences. (**C**) Quantitative chromatin immunoprecipitation (ChIP-qPCR) analyses using the indicated antibodies in *Aebp2* KO, *Aebp2* KO + L and *Aebp2* KO + S ESCs. Each experiment was performed at least three times on separate days. A representative experiment is shown. Error bars and the height of the bar show the standard deviation and the mean of three technical replicates, respectively. (**D**) Quantitative chromatin immunoprecipitation (ChIP-qPCR) analyses using the indicated antibodies in *Aebp2* KO, *Aebp2* KO + L and *Aebp2* KO + S ESCs. Each experiment was performed at least three times on separate days. A representative experiment is shown. Error bars and the height of the bar show the standard deviation and the mean of three technical replicates, respectively. (**E**) Violin plots representing the abundance of SUZ12 Rx-normalised reads per million ($log_2$RRPM) within 10 kb bins genome-wide (272,477 bins). Inside boxes represent the top quartile, the median and the bottom quartile.

© The Author(s)

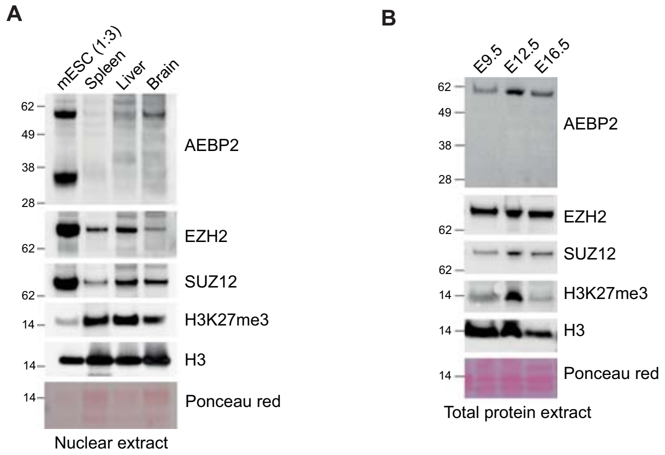


**Figure EV3. Expression of Aebp2$^{L}$ and Aebp2$^{S}$ in adults and during development.**

(**A**) Western blot analyses of proteins in nuclear extracts of mouse embryonic stem cells (mESCs), spleen, liver and brain, using the indicated antibodies. (**B**) Western blot analyses of proteins in whole-cell extracts from mice embryos from the indicated developmental stages, using the indicated antibodies.

 © The Author(s)

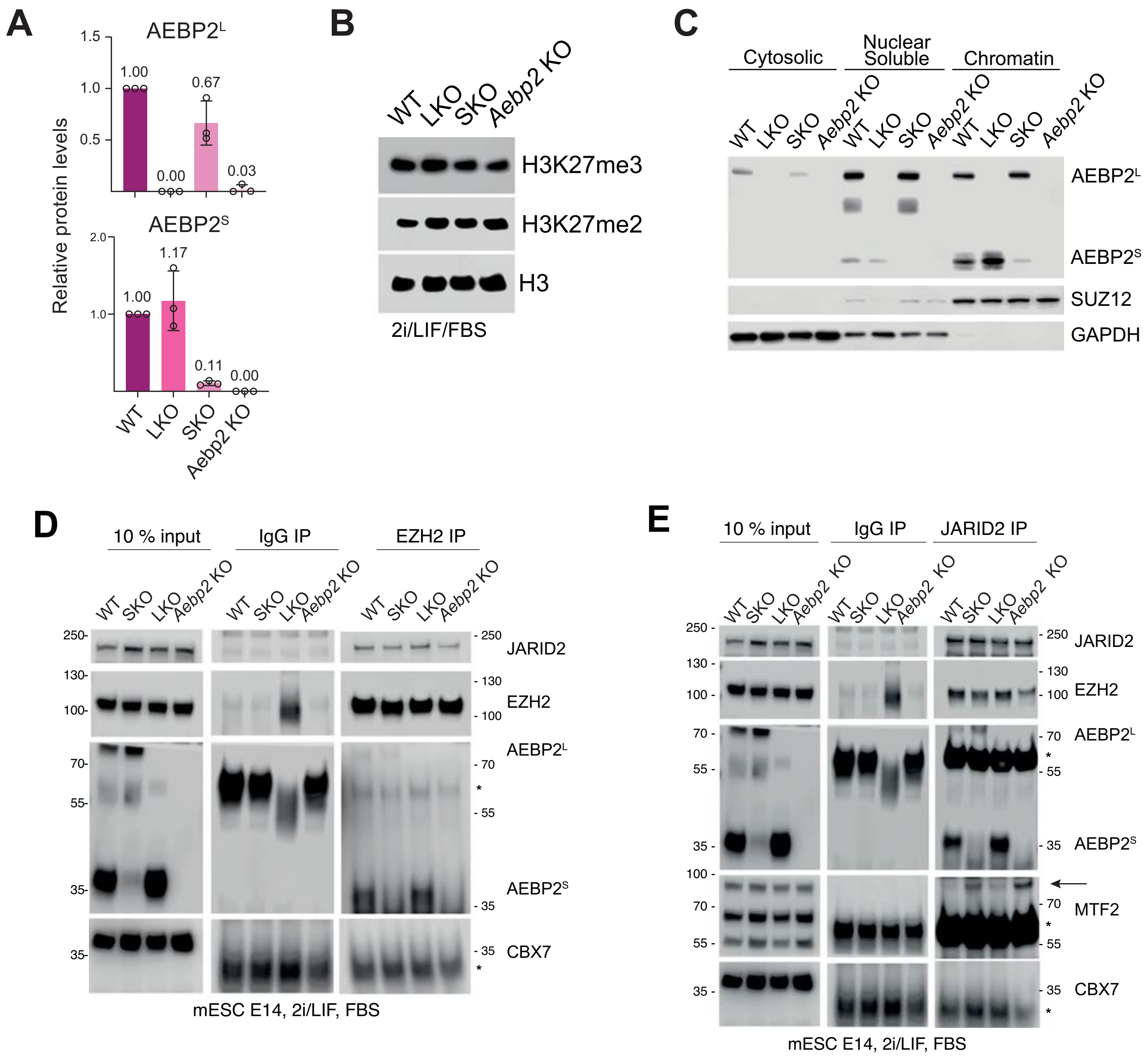


**Figure EV4. Characterisation of the composition of PRC2 in *Aebp2* KO ESC lines.**

(**A**) Quantification of AEBP2 expression in western blot analysis of whole-cell lysates. The ratio of AEBP2$^L$ and AEBP2$^S$ to normaliser band intensity in WT was set to 1. $n = 3$ biological independent samples. Data are shown as mean ± s.d. (**B**) Western blot analyses of H3K27me2 and H3K27me3 on whole-cell lysates from the indicated WT, LKO, SKO and *Aebp2* KO ESC lines. (**C**) Cellular fractionation of LKO, SKO, *Aebp2* KO, and matched WT ESCs, followed by western blot analyses, using the indicated antibodies. (**D**) Endogenous co-IPs of EZH2 in WT, LKO, SKO and *Aebp2* KO ESC lines, followed by Western blot analysis with the indicated antibodies. The 10% input and IgG IP lanes are identical to the ones from (**E**). (**E**) Endogenous co-IPs of JARID2 in WT, LKO, SKO and *Aebp2* KO ESC lines, followed by Western blot analysis with the indicated antibodies. The EZH2 and JARID2 co-IPs in (**D**, **E**) were simultaneously performed as part of one experiment. The 10% input and IgG IP lanes are identical to the ones from (**D**).

© The Author(s)

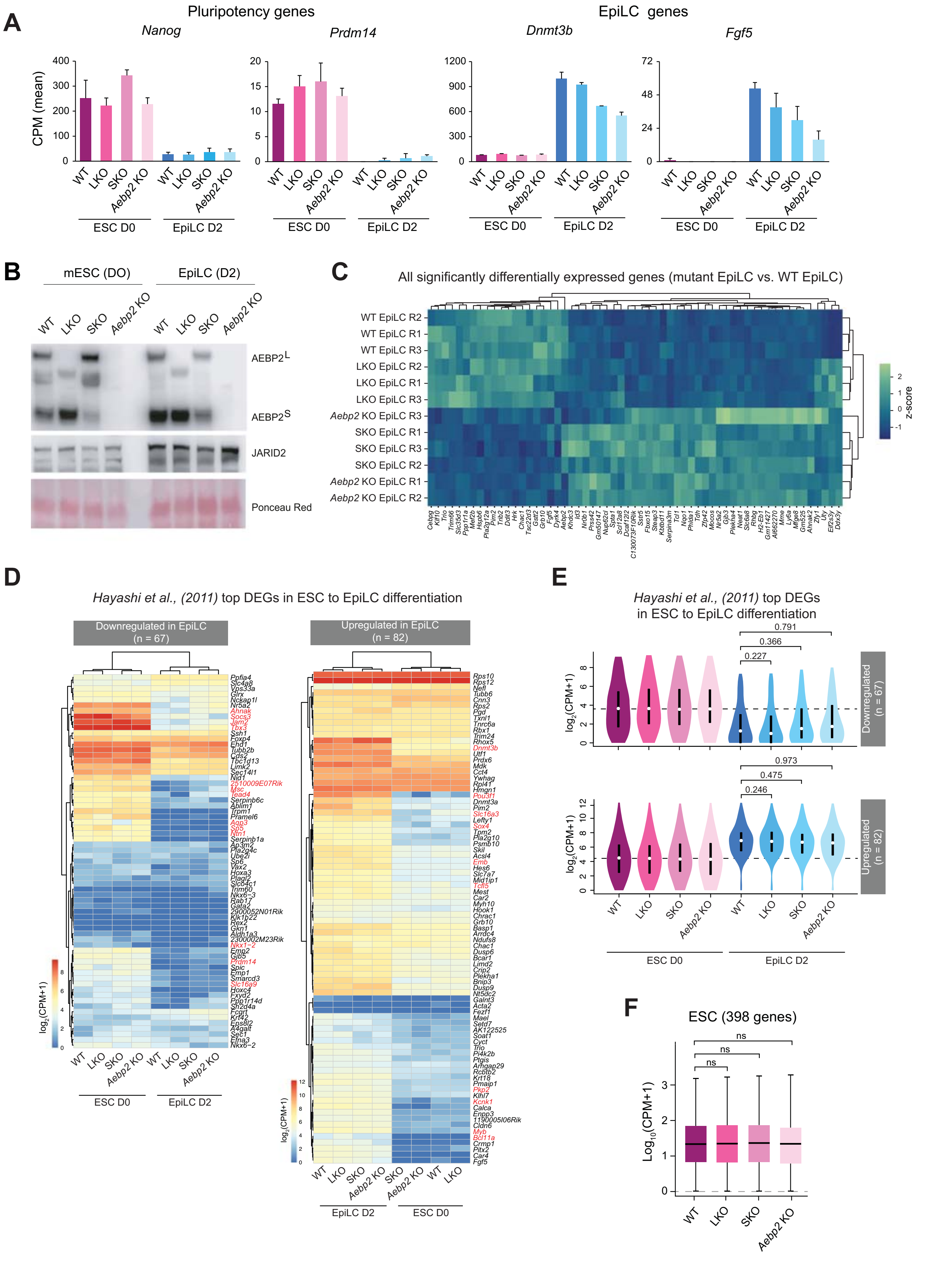
A
Pluripotency genes
EpiLC genes
Nanog
Prdm14
Dnmt3b
Fgf5
CPM (mean)
WT
LKO
SKO
Aebp2 KO
ESC D0
EpiLC D2
B
mESC (DO)
EpiLC (D2)
AEBP2L
AEBP2S
JARID2
Ponceau Red
C
All significantly differentially expressed genes (mutant EpiLC vs. WT EpiLC)
WT EpiLC R2
WT EpiLC R1
WT EpiLC R3
LKO EpiLC R2
LKO EpiLC R1
LKO EpiLC R3
Aebp2 KO EpiLC R3
SKO EpiLC R1
SKO EpiLC R3
SKO EpiLC R2
Aebp2 KO EpiLC R1
Aebp2 KO EpiLC R2
z-score
D
Hayashi et al., (2011) top DEGs in ESC to EpiLC differentiation
Downregulated in EpiLC (n = 67)
Upregulated in EpiLC (n = 82)
log2(CPM+1)
E
Hayashi et al., (2011) top DEGs in ESC to EpiLC differentiation
Downregulated (n = 67)
Upregulated (n = 82)
log2(CPM+1)
0.791
0.366
0.227
0.973
0.475
0.246
F
ESC (398 genes)
Log10(CPM+1)
ns

 © The Author(s)

**Figure EV5. Consequences of loss of AEBP2$^S$ or AEBP2$^L$ on EpiLC differentiation.**

(**A**) Bar plots showing the mean and standard deviation of relative expression of two representative pluripotency genes (*Nanog* and *Prdm14*) and two representative genes highly expressed at the EpiLC stage (*Fgf5* and *Dnmt3b*) at Day 0 and Day 2 timepoints, measured by Quant-Seq (CPM), based on three independent biological replicates performed on different days. (**B**) Western blot analyses using the indicated antibodies on whole-cell lysates from WT, LKO, SKO and *Aebp2* KO ESC lines, either before (mESCs) or after differentiation to EpiLC. (**C**) Heatmap showing unsupervised hierarchical clustering of the mRNA levels of significantly differentially expressed genes (DEG) at the EpiLC differentiation stage, between LKO vs. WT, SKO vs. WT, and *Aebp2* KO vs. WT, as measured by Quant-Seq. Scale: z-score. The gene lists can be found in Dataset EV4. (**D**) Heatmaps showing unsupervised hierarchical clustering of the mRNA levels of genes previously shown to be significantly differentially expressed during EpiLC differentiation (Hayashi et al, 2011), in WT, LKO, SKO and *Aebp2* KO cell lines at the two stages of differentiation, as measured by Quant-Seq. Data shown represent $\log_2$(mean CPM + 1) values of three independent biological triplicates. (**E**) Violin plots showing the mRNA levels of the genes from panel d. Shown is the mean of three independent biological triplicates. Wilcoxon test was used to measure statistical significance, with *P* values shown. The inside boxplots represent the interquartile range (Q1 to Q3), with a median indicated by the white dot. (**F**) Boxplots representing the mRNA levels of the 398 PRC2 de novo recruited genes at the ESC stage, measured by Quant-Seq. Shown is the mean of three independent biological replicates. Wilcoxon test was used to measure statistical significance. ns = not significant. The boxplots represent the interquartile range (Q1 to Q3), with a median indicated by the thick line, and the minimum and maximum values indicated by the whiskers.

© The Author(s)

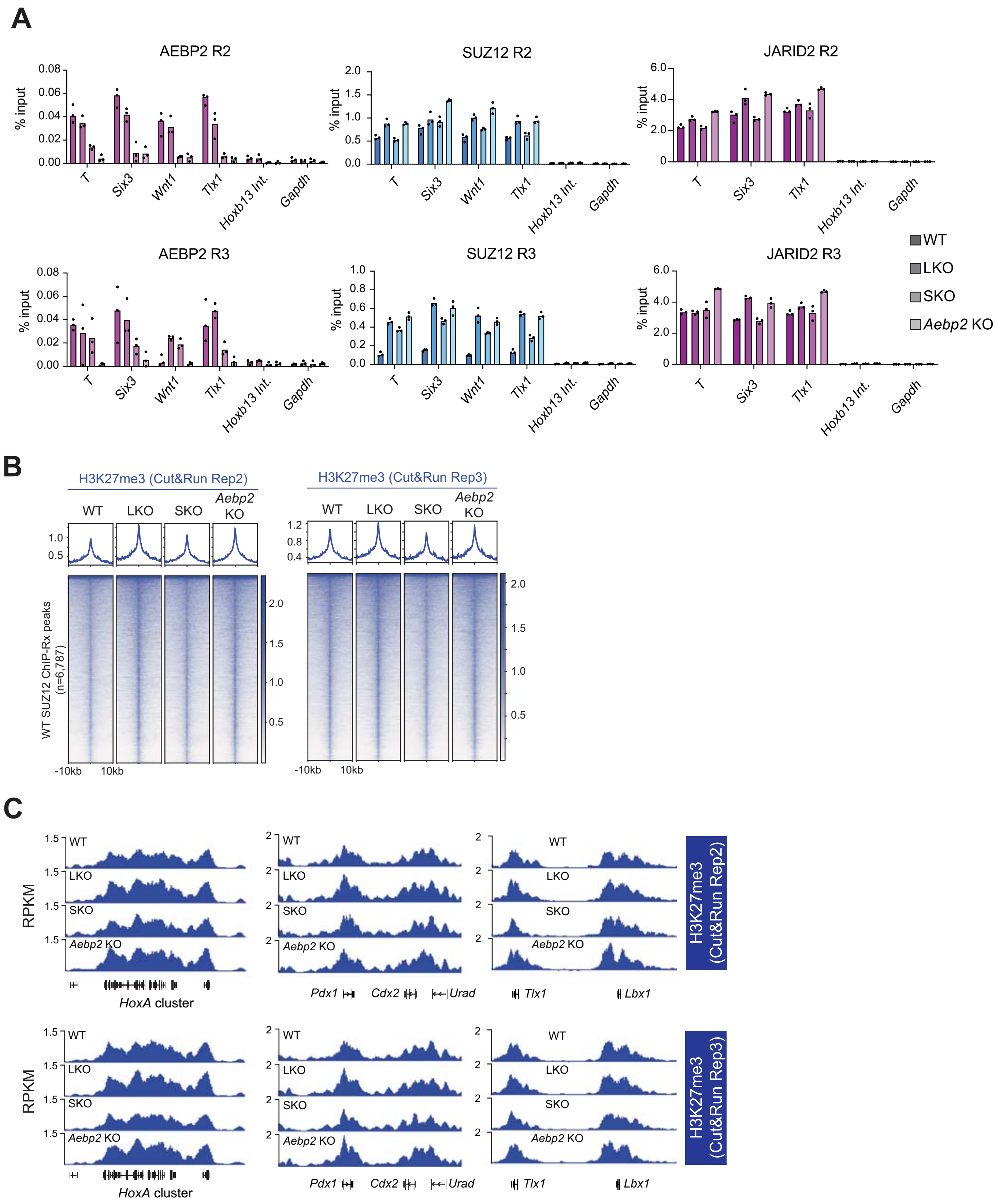
A
AEBP2 R2
SUZ12 R2
JARID2 R2
AEBP2 R3
SUZ12 R3
JARID2 R3
% input
T
Six3
Wnt1
Tlx1
Hoxb13 Int.
Gapdh
WT
LKO
SKO
Aebp2 KO
B
H3K27me3 (Cut&Run Rep2)
H3K27me3 (Cut&Run Rep3)
WT
LKO
SKO
Aebp2 KO
WT SUZ12 ChIP-Rx peaks (n=6,787)
-10kb
10kb
C
RPKM
HoxA cluster
Pdx1
Cdx2
Urad
Tlx1
Lbx1
H3K27me3 (Cut&Run Rep2)
H3K27me3 (Cut&Run Rep3)

 © The Author(s)

**Figure EV6. Loss of AEBP2$^L$ but not AEBP2$^S$ leads to increased PRC2 and H3K27me3 on Polycomb target genes in mouse ESCs.**

(**A**) Quantitative chromatin immunoprecipitation (ChIP) analyses using the indicated antibodies in WT, LKO, SKO and *Aebp2* KO ESCs. Two biological replicates are shown, independent of the ChIP-Rx replicate from Fig. 4. Error bars are representative of technical triplicates. (**B**) Average plot and heatmap representations of H3K27me3 CUT&RUN Replicates 2 and 3 RPKM values at WT SUZ12 peaks ($n = 6787$) in WT, LKO, SKO and *Aebp2* KO ESCs. Plots are centred on region midpoint $+-10$ kb. Relative intensities are indicated. (**C**) UCSC genome browser representations of H3K27me3 CUT&RUN Replicates 2 and 3 RPKM values at three representative PcG target loci (*HoxA*, *Pdx1/Cdx2, and Tlx1/Lbx1*) in WT, LKO, SKO and *Aebp2* KO cell lines.

© The Author(s) 

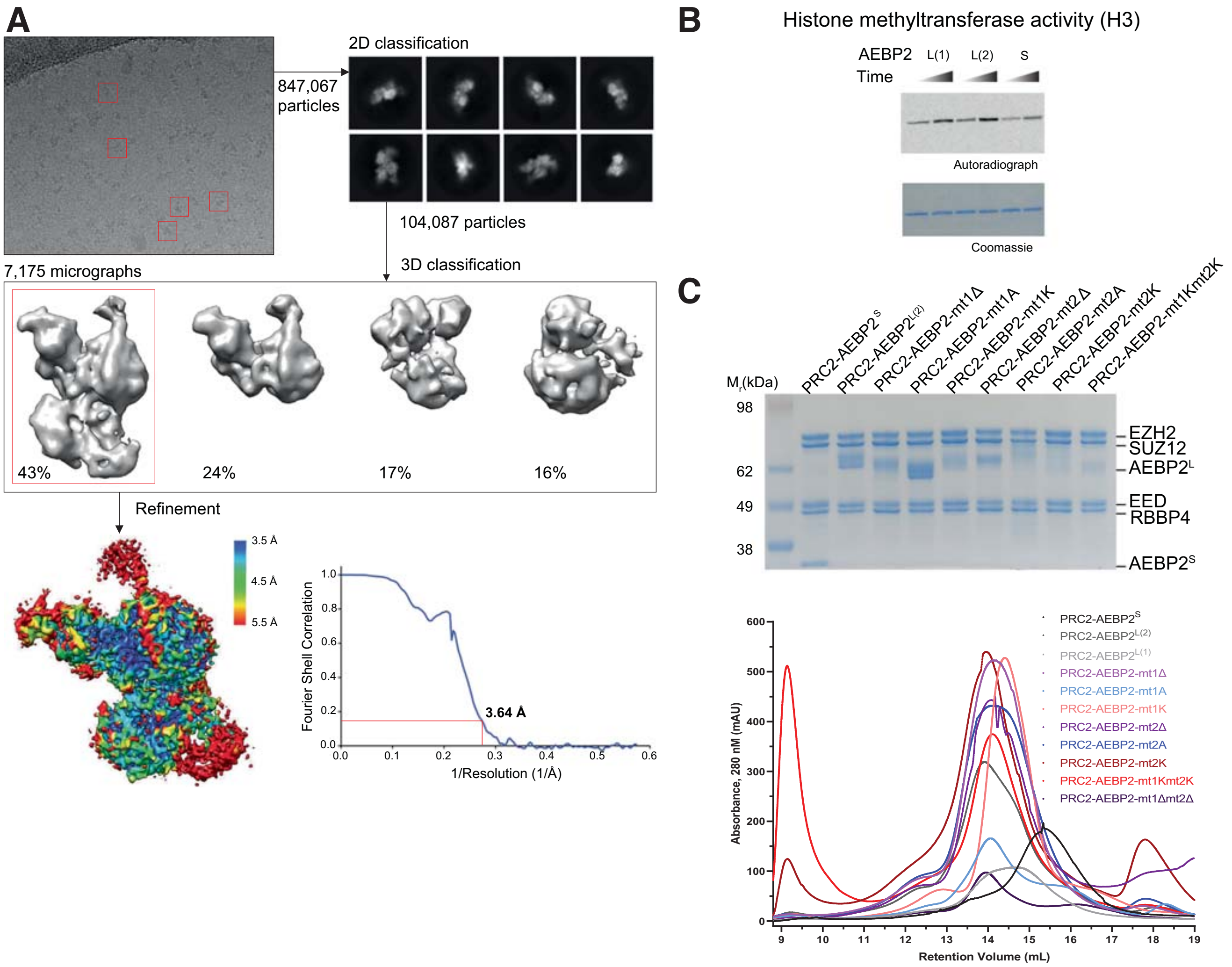


**Figure EV7. The N-terminal region of AEBP2$^{L}$ is disordered and utilises mammalian-specific acidic tracts to inhibit PRC2.**

(**A**) Cryo-EM processing workflow for PRC2–AEBP2$^{L}$. 847,067 particles were picked and sorted using two-dimensional classification. The resulting 104,087 particles were subjected to ab initio three-dimensional classification. This initial classification yielded one class with 45,024 particles that clearly corresponds to an intact complex. This subset was subjected to three-dimensional non-uniform refinement followed by global and local CTF refinement, yielding a 3.64 Å resolution cryo-EM map (gold-standard FSC = 0.143 criterion). Subsequent background subtraction and masked refinements of the individual domains and regions did not lead to map improvements. (**B**) A representative Coomassie blue-stained SDS–PAGE and the corresponding radiogram of the HMTase assay of AEBP2$^{L/S}$ with a H3.1-only substrate. (**C**) Coomassie blue-stained SDS–PAGE gel showing the purity of PRC2–AEBP2 complexes used for Fig. 5G and (**B**), and well as representative gel filtration chromatograms (Superose 6 Increase 10/300 GL) of proteins used for Fig. 5, and this figure.

 © The Author(s)

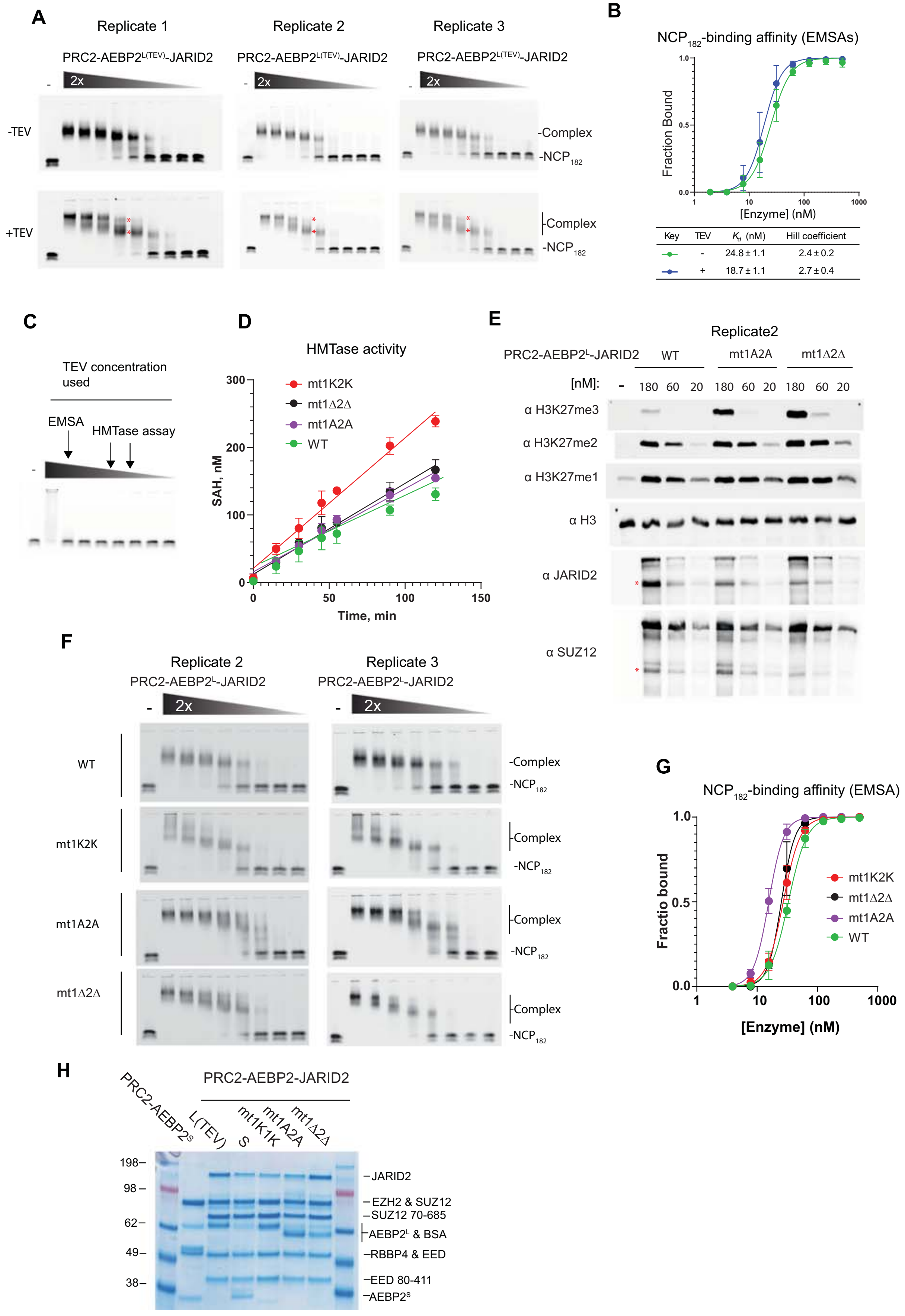
A
Replicate 1
Replicate 2
Replicate 3
PRC2-AEBP2L(TEV)-JARID2
2x
-TEV
+TEV
-Complex
-NCP182
B
NCP182-binding affinity (EMSAs)
Fraction Bound
[Enzyme] (nM)
Key TEV Kd (nM) Hill coefficient
- 24.8 ± 1.1 2.4 ± 0.2
+ 18.7 ± 1.1 2.7 ± 0.4
C
TEV concentration used
EMSA
HMTase assay
D
HMTase activity
mt1K2K
mt1Δ2Δ
mt1A2A
WT
SAH, nM
Time, min
E
Replicate2
PRC2-AEBP2L-JARID2 WT mt1A2A mt1Δ2Δ
[nM]: - 180 60 20 180 60 20 180 60 20
α H3K27me3
α H3K27me2
α H3K27me1
α H3
α JARID2
α SUZ12
F
Replicate 2
Replicate 3
PRC2-AEBP2L-JARID2
WT
mt1K2K
mt1A2A
mt1Δ2Δ
-Complex
-NCP182
G
NCP182-binding affinity (EMSA)
Fractio bound
[Enzyme] (nM)
H
PRC2-AEBP2-JARID2
PRC2-AEBP2S
L(TEV)
S
mt1K1K
mt1A2A
mt1Δ2Δ
198–
98–
62–
49–
38–
–JARID2
–EZH2 & SUZ12
–SUZ12 70-685
AEBP2L & BSA
–RBBP4 & EED
–EED 80-411
–AEBP2S

© The Author(s) 

**Figure EV8. AEBP2$^L$ antagonises PRC2.2.**

(**A**) PRC2-AEBP2$^{L(TEV)}$-JARID2 was treated in the presence or absence of TEV protease before were subjected to EMSA with 5 nM Cy5-NCP$_{182}$ probe. The protein was subjected to a twofold serial dilution starting from 500 nM. Red asterisks indicate multiple bound complexes that are migrated with different velocities. (**B**) Top: binding curves of the EMSA from (**A**). Bottom: The derived $K_d$ and Hill coefficients. Means and error bars represent the average SAH concentration and the standard error, respectively, based on three independent replicates performed on 3 different days. (**C**) To identify a TEV concentration that would not interfere with a binding assay, EMSA was carried out using 5 nM Cy5-NCP$_{182}$ probe and twofold serially diluted TEV protease, starting from 0.4 mg/ml. "TEV concentration used" indicated the TEV concentration that was selected for EMSA experiments (0.2 mg/ml) in (**A**) and final TEV concentrations in the HMTase reactions in Fig. 6C that were carried out in the presence of 50 nM and 20 nM PRC2-AEBP2$^{L(TEV)}$-JARID2 (TEV concentration of 0.02 mg/ml and 0.008 mg/ml, respectively). (**D**) Progress curves were carried out under the same conditions used for the Michaelis–Menten kinetic analysis in Fig. 6F, except that 1 μM chromatin (NCP equivalent) and 25 nM PRC2-AEBP2$^L$-JARID2 complexes as indicated were used. The reaction was stopped at 15 min, 20 min, 45 min, 55 min, 90 min and 120 min before the produced SAH was quantified. Mean are derived from two independent experiments that were carried out on different days and the error bars represent standard deviation. A linear regression was used to fit a linear line across the means. (**E**) Second replicate of the immunoblotting from Fig. 6F, using antibodies as indicated. Red asterisks indicate degradation products of SUZ12 and JARID2. (**F**) Second and third replicates of the EMSA shown in Fig. 6G. (**G**) Binding curves of different PRC2-AEBP2$^L$-JARID2 complexes, as indicated, and NCP$_{182}$ nucleosomal probe. Means represent the fraction of the bound probe as quantified from the three independent EMSA experiments presented in Fig. 6G and in (**F**), and the error bars represent standard deviation. (**H**) SDS–PAGE analysis of the sample used for fluorescence anisotropy in Fig. 6J. 8 μL of the binding reaction from the highest protein concentration (500 nM) was mixed with 4 μL of 4X LDS buffer and subjected to SDS–PAGE. "S" indicates a TEV-free PRC2-AEBP2$^S$-JARID2, generated by the TEV cleavage of PRC2-AEBP2$^{L(TEV)}$-JARID2 followed by the removal of the TEV enzyme using size exclusion chromatography.

 © The Author(s)

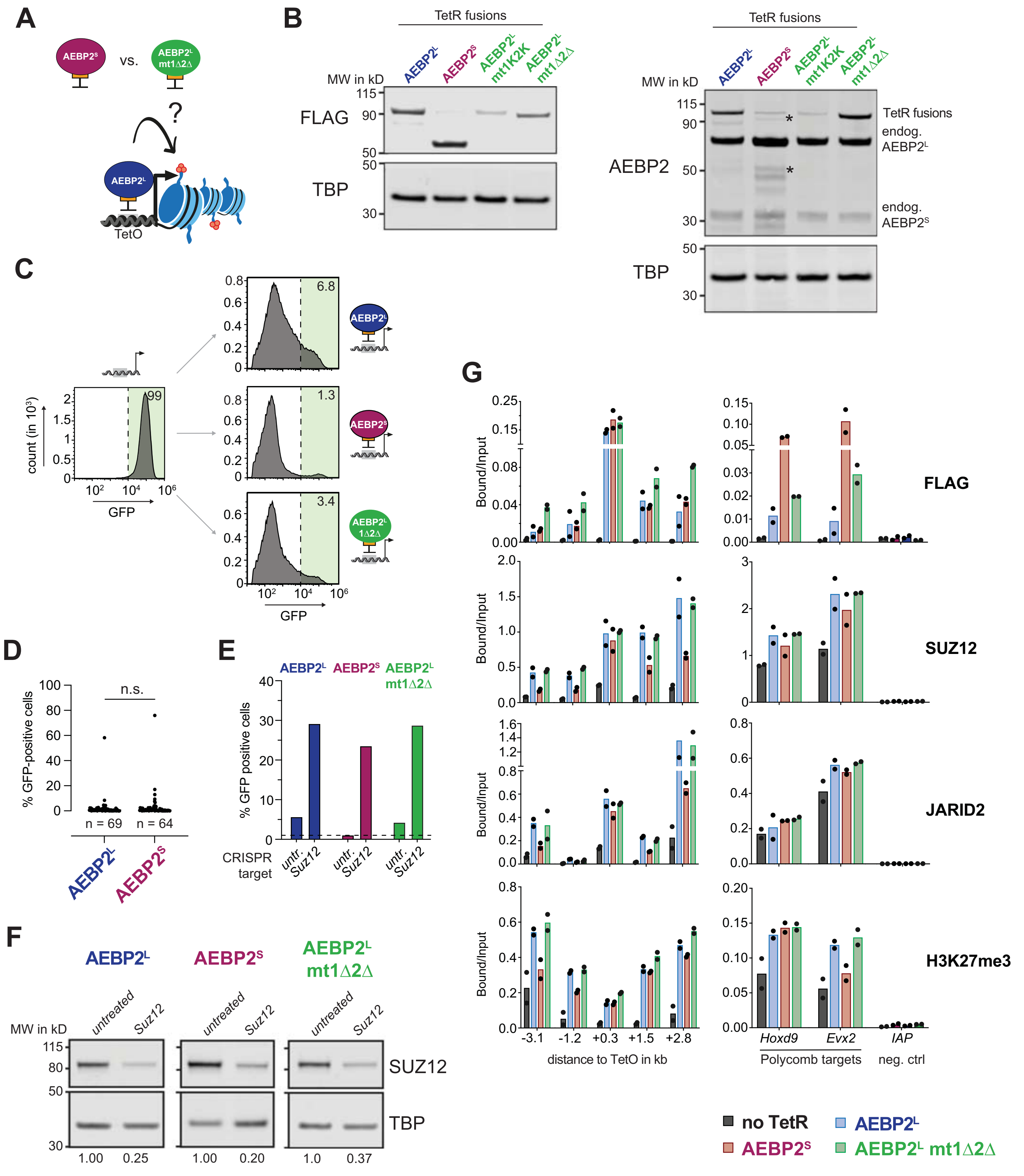
A
AEBP2S
vs.
AEBP2L mt1Δ2Δ
?
AEBP2L
TetO
B
TetR fusions
MW in kD
AEBP2L
AEBP2S
AEBP2L mt1K2K
AEBP2L mt1Δ2Δ
FLAG
TBP
115
90
50
30
AEBP2
TetR fusions
endog. AEBP2L
endog. AEBP2S
*
C
count (in 10³)
GFP
99
6.8
1.3
3.4
10²
10⁴
10⁶
D
% GFP-positive cells
n.s.
n = 69
n = 64
AEBP2L
AEBP2S
E
% GFP positive cells
AEBP2L AEBP2S AEBP2L mt1Δ2Δ
CRISPR target
untr.
Suz12
F
AEBP2L
AEBP2S
AEBP2L mt1Δ2Δ
MW in kD
untreated
Suz12
SUZ12
TBP
1.00 0.25
1.00 0.20
1.0 0.37
G
Bound/Input
FLAG
SUZ12
JARID2
H3K27me3
-3.1 -1.2 +0.3 +1.5 +2.8
distance to TetO in kb
Hoxd9
Evx2
IAP
Polycomb targets
neg. ctrl
no TetR
AEBP2L
AEBP2S
AEBP2L mt1Δ2Δ

© The Author(s)

**Figure EV9. The antagonistic effect of AEBP2$^L$ can be rescued by heterologous tethering to chromatin.**

(**A**) Scheme of experimental design. TetR fusions facilitate forced recruitment of wild-type and mutant AEBP2 isoforms to Tet Operator sites (TetO) upstream of a reporter gene to compare the impact on chromatin modifications and gene expression. (**B**) Immunoblots show expression levels of TetR fusion proteins relative to endogenous AEBP2 isoforms. TBP serves as loading controls. Asterisks indicate residual uncleaved mCherry-P2A-TetR fusion proteins (**C**) Flow cytometry histograms of GFP expression in the absence (left) and presence of TetR fusions with AEBP2 isoforms (right). Inserted numbers indicate the percentage of GFP-positive reporter ESCs. (**D**) Quantification of percentage of GFP-positive cells in clonal reporter ESC lines in expressing AEBP2$^L$ or AEBP2$^S$. (**E**) Bar plot shows the percentage of GFP-positive TetR-AEBP2$^L$ or TetR-AEBP2$^S$ reporter ESCs following transduction with Cas9/sgRNAs targeting *Suz12*. Untransduced reporter ESCs serve as controls ("untr."). (**F**) Immunoblotting of the mESC reporter cell lines that were assayed in (**E**) using antibodies as indicated. (**G**) ChIP-qPCR analysis shows relative enrichments of FLAG-TetR fusions, Polycomb proteins and histone modifications upstream and downstream of TetO (left) and at endogenous loci (right) in reporter ESCs. Shown are data of two independent experimental replicates.

 © The Author(s)
